# Minimal important change in physical function in trauma patients: a study using the short musculoskeletal function assessment

**DOI:** 10.1007/s11136-020-02476-8

**Published:** 2020-04-04

**Authors:** M. W. de Graaf, I. H. F. Reininga, E. Heineman, M. El Moumni

**Affiliations:** 1 Department of Trauma Surgery, University Medical Center Groningen, University of Groningen, PO Box 30 001, 9700 RB Groningen, The Netherlands; 2 Department of Surgery, University Medical Center Groningen, University of Groningen, PO Box 30 001, 9700 RB Groningen, The Netherlands

**Keywords:** Short musculoskeletal function assessment, Patient reported outcome, Minimal important difference, Minimal clinically important, Trauma, Injury

## Abstract

**Purpose:**

The Short Musculoskeletal Function Assessment (SMFA) questionnaire can be used to evaluate physical functioning in patients with traumatic injuries. It is not known what change in score reflects a meaningful change to patients. The aim was to determine minimal important change (MIC) values of the subscales (0–100) of the Dutch SMFA-NL in a sample of patients with a broad range of injuries.

**Methods:**

Patients between 18 and 65 years of age completed the SMFA-NL and the Global Rating of Effect (GRE) questions at 6-week and 12-month post-injury. Anchor-based MIC values were calculated using univariable logistic regression analyses.

**Results:**

A total of 225 patients were included (response rate 67%). The MIC value of the *Upper Extremity Dysfunction* (UED) subscale was 8 points, with a misclassification rate of 43%. The *Lower Extremity Dysfunction* subscale MIC value was 14 points, with a misclassification rate of 29%. The MIC value of the *Problems with Daily Activities* subscale was 25 points, with a misclassification rate of 33%. The MIC value of the *Mental and Emotional Problems* (MEP) subscale was 7 points, with a misclassification rate 37%.

**Conclusion:**

MIC values of the SMFA-NL were determined. The MIC values aid interpreting whether a change in physical functioning can be considered clinically important. Due to the considerable rates of misclassification, the MIC values of the UED and MEP subscales should be used with caution.

## Introduction

The Short Musculoskeletal Function Assessment (SMFA) is a patient-reported outcome measure (PROM) that can be used to evaluate physical functioning in patients that sustained traumatic injuries [1, 2]. Repeated assessment of patients’ physical function is an important aspect of the treatment of trauma patients. Knowledge of what change in function is meaningful to patients, may help clinicians to interpret improvement at an outpatient clinic visit, or may help the interpretation of clinical research when the SMFA is used as functional outcome [3]. To evaluate a patient’s recovery, changes in score may be tested for statistical significance. However this does not necessarily mean the change is meaningful, since it does not directly relate to what patients consider an important change.

Interpretability is the degree to which one can assign qualitative meaning (i.e., clinical or commonly understood connotations) to an instrument’s quantitative scores or change in scores [4]. Though interpretability is not a measurement property of a PROM-like validity and reliability, it is an important aspect for a proper use of a measurement instrument [5, 6]. Normative data facilitate the interpretability of quantitative scores, by providing a reference of which scores represent a ‘normal’ level of functioning [6, 7]. However, normative data do not relate to what *change* in score can be considered meaningful. The smallest change in score that is considered an *important* change by patients, has been defined as the Minimal Important Change (MIC) [4, 5]. Although the SMFA is a widely used questionnaire, MIC values have never been reported for it [2]. Thus, it is not known what change in SMFA scores can be considered meaningful.

Methods to estimate what change in score can be considered clinically important, can coarsely be divided into two groups: distribution-based and anchor-based methods [8, 9]. Distribution-based methods solely rely on mathematical parameters, for example, by relating change to the standard deviation [9, 10]. However, distribution-based methods do not directly relate to what patients consider an important change [8, 9]. Anchor-based methods use an external criterion, to evaluate whether the change in score was perceived as important by the patient, and have been advocated as a more appropriate method to obtain MIC values [3, 6, 8, 11].

In this study we were specifically interested in what *patients* perceive as an important change in physical function. Therefore, the aim was to determine anchor-based MIC values for the subscales of the Dutch SMFA-NL, in a sample of patients with a broad range of traumatic injuries.

## Materials and methods

### Study design

A longitudinal cohort study design was used, in which patients received questionnaires at 6-week and 12-month post-injury. Patients were recruited at the trauma department of the University Medical Center Groningen (level 1 trauma center, The Netherlands). Patients provided oral or written consent before they participated in this study. The methods employed in this study have been reviewed by the local Institutional Review Board (METC University Medical Center Groningen), which waived further need for approval (METc2012.104) because the study did not fall under the Dutch WMO law. The study was carried out in compliance with the principles outlined in the Declaration of Helsinki [12].

### Patients

Patients that presented with one or more acute traumatic injuries, that required follow-up treatment at the trauma surgery outpatient clinic, were prompted for participation. Patients had a broad range of acute traumatic injuries, ranging from soft-tissue injury to multiple injuries after high-energy trauma, that were treated either surgically or conservatively. Exclusion criteria were as follows: age under 18 or above 65 years, being unable to read and write Dutch, fractures that resulted in severe neurological deficits, severe traumatic brain injury, pathologic fractures and patients that had severe psychiatric or cognitive conditions.

### Questionnaires

#### The short musculoskeletal function assessment

The SMFA questionnaire has been developed by Swiontkowski et al. and was designed to assess physical functioning in patients with a broad range of musculoskeletal disorders, including injuries [1]. The SMFA contains 46 items that are scored on a five-point Likert scale. The Dutch SMFA-NL consists of four subscales: *Upper Extremity Dysfunction, Lower Extremity Dysfunction, Problems with Daily Activities* and *Mental and Emotional Problems* [13, 14]. Scores range from 0 to 100. A score of 0 represents best possible function. Patients received the standard SMFA-NL. The SMFA-NL uses the same items as the original American SMFA, but uses a different factor structure [14]**.** The clinimetric properties of the SMFA-NL have been shown to be sufficient in patients with traumatic injuries [14, 15].

#### Global rating of effect questions

Global rating of effect (GRE) questions were used as external anchor, to evaluate whether patients had improved, deteriorated or had not changed, relative to the 6-week post-injury measurement. GRE questions were constructed for each of the constructs that were assessed with the SMFA-NL (Appendix 1). The GRE questions were formulated as in the following example: “How much problems do you currently have with performing your daily activities (such as self-care, doing groceries, labor, hobbies or household tasks), in comparison with 6 weeks after the injury?” Patients could choose from five possible answers: “much improved,” “slightly improved,” “about the same,” “slightly deteriorated,” and “much deteriorated.”

### Procedures

Patients that participated in the study received the SMFA-NL questionnaire at 6-week post-injury and received the follow-up questionnaires (SMFA-NL and GRE) at 12-month post-injury. Patients received the questionnaires per mail or electronically. Non-responders were reminded once.

### Sample size

According to the COSMIN guidelines, at least 50 patients that have changed and 50 patients that have not changed should be included [5, 16]. We hypothesized that 75% of the patients would report improvement and 25% would report ‘no change’ in physical function relative to the 6-week post-injury measurement. Therefore, the aim was to include at least 200 patients so that at least 50 patients per group (importantly improved and not-importantly changed) were included.

### Data analysis

The data of patients that completed the 6-week post-injury measurement and the 12-month post-injury measurement were used in this study. Frequencies and proportions were calculated for categorical variables such as gender, educational level, marital status, chronic health conditions and anatomic location of the injury. The Injury Severity Score (ISS) in the sample was calculated as median (IQR) [17]. The change in SMFA-NL subscale scores between the 6-week and 12-month post-injury measurements were calculated.

A dichotomous anchor was constructed using the GRE questions, dividing patients that reported no change and patients that importantly improved. Those that answered “slightly improved,” “about the same,” or “slightly deteriorated” were considered *unchanged*. Patients that answered the subscale-specific GRE as “much improved” were considered *importantly improved*. An anchor for important deterioration was not created.

The presence of floor and ceiling effects was evaluated. A floor or ceiling effect was considered present, if 15% or more of the patients reported the highest or lowest possible score at 6-week post-injury. Patients without upper or lower extremity injuries may be expected to report the best possible score on the *Upper* or *Lower Extremity Dysfunction* subscales, respectively. Hence, floor and ceiling effects on the *Upper* and *Lower extremity* subscales were analyzed in patients that had an upper or lower extremity injury, respectively. The whole study sample was used to analyze floor and ceiling effects with regard to the *Problems with Daily Activities* and *Mental and Emotional Problems* subscales.

### Statistical analysis

The MIC values were calculated using the predictive modeling approach of Terluin et al. [18]. A univariable logistic regression was performed for each subscale of the SMFA-NL. Outcome variable was the created dichotomous anchor (importantly improved, and unchanged patients). The predictor variable was the change in SMFA-NL score on the specific subscale. To reduce bias due to unequal proportions of improved and unimproved patients, the predicted MIC (MIC_pred_) values were adjusted as described by Terluin et al., resulting in MIC_adj_ values [19]. For a further specification of the predictive modeling approach and adjustment for unequal proportions, we refer to the original work of Terluin et al. [18, 19]. A figure was constructed in which the MIC values were shown in respect to the smallest detectable change (SDC) of the SMFA-NL [20]. The SDC denotes the smallest change in score that is not due to measurement error. The SDC of the SMFA-NL has been calculated for trauma patients in previous research and ranged from 11.0 to 17.4 [15].

Since the anchor served as a gold standard to determine the MIC value, its ‘diagnostic performance’ was evaluated. For each MIC value, the sensitivity, specificity, percentage of misclassification and Area Under the Curve (AUC) calculated.

Because the SMFA-NL evaluates physical functioning of the entire human body, patients answered the items of the *Lower Extremity Dysfunction* subscale even if they did not have a lower extremity injury. The same counts for the *Upper Extremity Dysfunction* subscale. A subgroup analysis was performed in which MIC values of the *Upper Extremity Dysfunction* and *Lower Extremity Dysfunction* subscales were calculated only for patients that had at least one injury of the upper or lower extremity, respectively.

A sensitivity analysis was performed in which only patients that reported to be ‘about the same’ were considered unchanged. Patients that reported ‘slightly improved’ were considered to be importantly improved. MIC _pred_, MIC_adj_ and parameters of diagnostic accuracy were reported. Missing data were handled listwise. Statistical analyses were performed in IBM SPSS statistics for Windows, Version 23.0, Armonk, NY: IBM corp.

## Results

### Sample characteristics

A total of 513 patients completed the SMFA-NL at 6-week post-injury (response rate: 67%). A total of 225 patients filled in the 12-month post-injury questionnaire. The general characteristics of the study sample are shown in Table 1. The sample consisted of 130 (58%) males and 95 (42%) females. Upper or lower extremity injuries were most prevalent (Table 1). Patients had a median ISS of 4 points.

**Table 1:** General characteristics

	*N* (%)
Gender (*n* = 225)	
Male	130 (58)
Female	96 (42)
Age (*n* = 225)^a^	47 (13)
Marital status (*n* = 212)	
Single	74 (35)
With partner	138 (65)
Educational level (*n* = 211)	
Elementary school	4 (2)
High school	69 (33)
College	60 (27)
Bachelors degree or higher	77 (36)
Other	1 (0)
Chronic health conditions (*n* = 207)	
None	114 (55)
One or two	74 (36)
Three or more	19 (9)
Injuries (*n* = 500)	
Head and neck	40 (8)
Face	21 (4)
Thorax	45 (9)
Abdomen	18 (4)
Extremities	
Upper extremity	151 (30)
Lower extremity	167 (33)
External^b^	60 (12)
Injury severity score^c^	4 (5)

^a^Mean (SD)

^b^External includes contusions, distortions, skin lacerations and whole body injuries

^c^Median (IQR)

The change in SMFA-NL subscale score per GRE category is shown in Table 2. ‘Strong improvement’ was reported most frequently among all subscales. ‘Much deteriorated’ was reported least. The *Upper Extremity Dysfunction* subscale showed a floor effect: 26 (23%) of the patients with an upper extremity injury reported a lowest possible score at 6-week post-injury. Floor or ceiling effects were not observed on the other subscales.

**Table 2:** Change in SMFA-NL subscale per GRE category

Global rating of effect	Upper extremity dysfunction		Lower extremity dysfunction		Problems with daily activities		Mental and emotional problems	
	*n*	Mean (SD)	*n*	Mean (SD)	*n*	Mean (SD)	*n*	Mean (SD)
Strong improvement	116	12.5 (18.2)	104	25.6 (21.3)	121	35.5 (21.3)	59	9.5 (10.8)
Slight improvement^a^	28	14.7 (23.1)	20	11.8 (14.8)	34	23.0 (16.2)	51	4.0 (13.3)
About the same^a^	61	1.4 (7.4)	65	5.5 (11.9)	27	16.8 (22.3)	78	1.0 (10.6)
Slight deterioration^a^	11	6.4 (16.7)	9	− 3.7 (13.0)	13	8.3 (24.1)	21	− 5.1 (10.3)
Strong deterioration	3	− 12.5 (23.2)	7	12.2 (22.3)	9	9.3 (25.2)	5	− 19.3 (27.9)

^a^Groups that together form the “unchanged” patients in the anchor

### Minimal important change

**Fig. 1:**
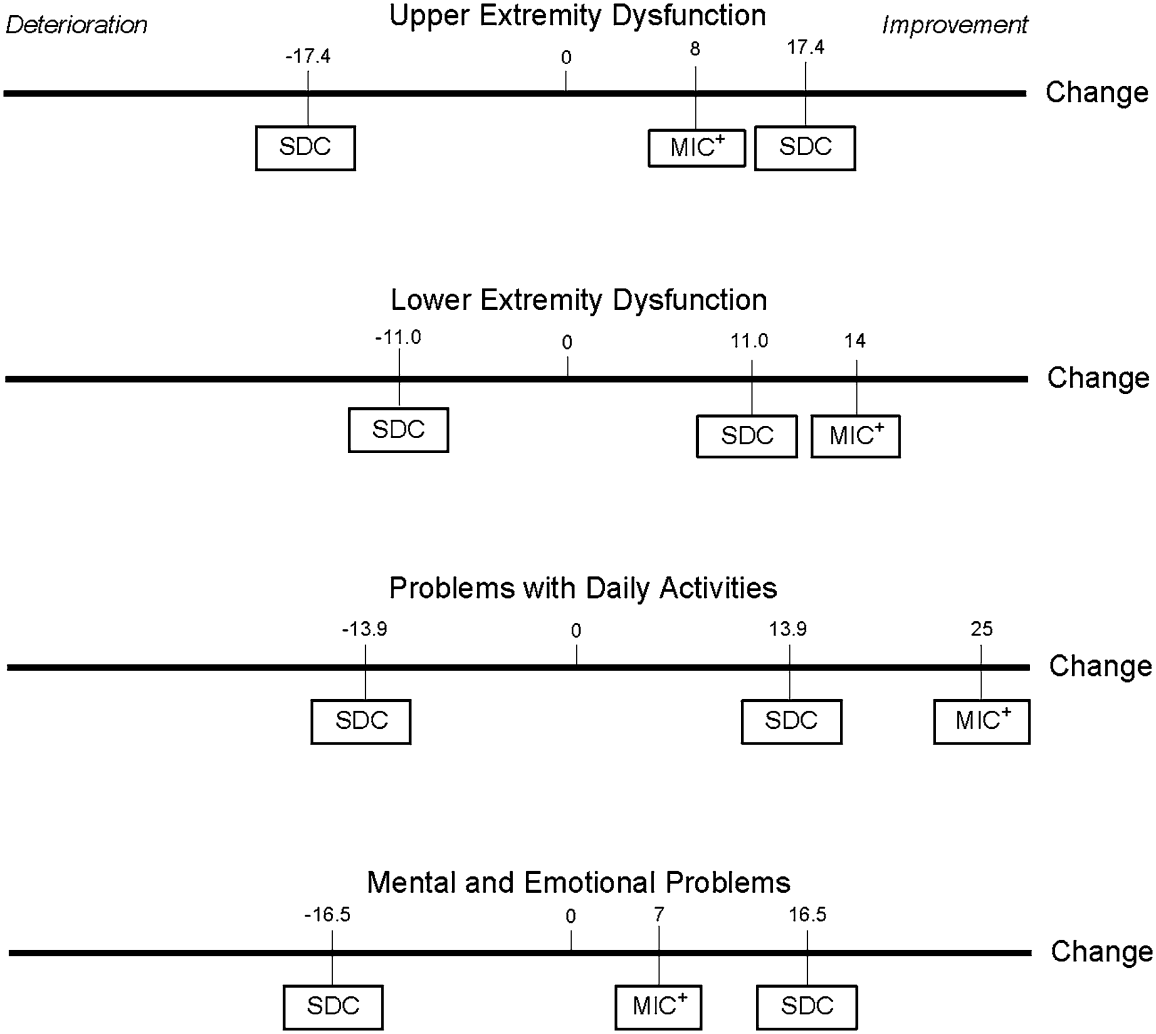
Minimal important change values for the subscales of the SMFA-NL. A visual representation of the minimal important change (MIC) and smallest detectable change (SDC) values within and between the subscales of the SMFA-NL. The MIC and SDC values were plotted on an axis that represents change in SMFA-NL score in points. MIC values on the left side of 0 were not plotted, because a MIC for deterioration was not calculated

The predicted and adjusted MIC values are shown in Table 3. The MIC_adj_ value of the *Upper Extremity Dysfunction* subscale was 8 points, with a misclassification rate of 43%. The *Lower Extremity Dysfunction* subscale MIC_adj_ value was 14 points, with a misclassification rate of 29%. The MIC_adj_ value of the *Problems with Daily Activities* subscale was 25 points, with a misclassification rate of 33%. The MIC_adj_ value of the *Mental and Emotional Problems* subscale was 7 points with a misclassification rate 37%. The corresponding sensitivity, specificity and AUC are shown in Table 3. The MIC values are visualized in Fig. 1.

**Table 3:** Minimal important change scores for improvement

SMFA-NL subscales	*N*	Prop.Impr.	Corr	MIC pred	95% CI	MIC_adj_	Sens (%)	Spec (%)	%Mis	AUC
Upper extremity dysfunction	216	0.53	0.20	9	− 5.1–23.5	8	66	53	43	0.63
≥ One upper extremity injury	108	0.66	0.13	17	N.a	15	65	38	53	0.59
Lower extremity dysfunction	198	0.53	0.47	14	9.4–20.0	14	62	82	29	0.78
≥ One lower extremity injury	89	0.72	0.37	26	14.8–42.2	24	69	72	30	0.74
Problems with daily activities	195	0.62	0.37	26	18.0–35.6	25	66	69	33	0.72
Mental and emotional problems	209	0.72	0.31	5	− 0.45–10.5	7	64	63	37	0.69

Adjusted minimal important change values of the SMFA-NL subscales were shown. All subscales use a 0–100 scale, in which 0 is best

*Prop. impr*. proportion of patients reporting improvement, *Corr* correlation of the anchor to the change-score, *MIC*_*pred*_ minimal important change for improvement, *MIC*_*adj*_ minimal important change for improvement adjusted for unequal proportions of improved/unchanged patients, *Sens* sensitivity, *spec* specificity, *%Mis* percentage misclassification *AUC* area under the curve, *N.a*. not available, interval was too wide

#### Subgroup analysis

In the subgroup of patients with at least one upper extremity injury, the MIC value of the *Upper Extremity Dysfunction* subscale was 15 points. The MIC value of the *Lower Extremity Dysfunction* subscale was 24 points for patients with at least one lower extremity injury.

#### Sensitivity analysis

The MIC_adj_ value of the *Upper and Lower Extremity Dysfunction* subscales were 4 and 11 points, respectively. The MIC_adj_ value of the *Problems with Daily Activities* subscale was 20 points. The MIC_adj_ value of the *Mental and Emotional Problems* subscale was 4 points. The corresponding sensitivity, specificity, AUC and misclassification rates are shown in Appendix 2.

## Discussion

Anchor-based minimal important change values were identified for all subscales of the SMFA-NL in patients that sustained an acute traumatic injury. The MIC_adj_ values ranged from 8 to 25 points. The MIC value of the *Upper Extremity Dysfunction* subscale was associated with the highest misclassification rate. The MIC value of the *Lower Extremity Dysfunction* subscale was associated with the lowest misclassification rate.

Monitoring functional recovery is a keystone of the treatment of patients with injuries. Determining what difference is clinically relevant is important. Because small differences in mean health-related quality-of-life score may yield statistically significant differences when a sample is large, but statistical significance is not equal to clinical relevance. The MIC values provided in the present study can be used to evaluate whether the occurred change in physical function can be considered clinically relevant.

The sensitivity analysis showed that minimally important changes were smaller when ‘slightly improved’ patients were considered ‘importantly improved.’ There is no consensus whether ‘slightly improved’ patients should be considered ‘importantly improved’ or ‘unchanged.’ The number of ‘unchanged’ patients in the *Problems with Daily Activities* was smaller than recommended to evaluate MIC values, and therefore should be interpreted with caution [5, 16].

In contrast to the full sample, the subgroups were more homogeneous in terms of injury type (i.e., the *Upper Extremity Dysfunction* MIC value was calculated for patients with least one upper extremity injury). The physical functioning of the extremity was expected to be more strongly affected in the subgroup, resulting in higher MIC values. This suggests that patients with a stronger affected extremity, required a larger change in score to be considered important. This is in line with previous studies. De Vet et al. showed that a more severe condition, requires a greater improvement in score to be considered important to patients, yielding a larger MIC value [21].

To the best of our knowledge, an anchor-based MIC value has not been reported for the SMFA. Studies that related clinical importance to the scores of the SMFA all used distribution-based methods. Hedbeck et al. evaluated minimally important change of the SMFA in 120 elderly with an acute displaced femoral neck fracture [22]. The change in SMFA score was related to the Harris Hip Score, which was used as an external criterion. However, a specific MIC value was not reported [22]. Hedbeck et al. defined important change using the distribution-based 0.5 SD method, which as been suggested to represent an estimate of clinical importance [10]. However the 0.5 SD method, and all other distribution-based methods, define clinical importance solely from statistical parameters, and do not relate to what patients actually consider an important change [8, 11].

In two other studies, minimal important difference (MID) values were reported for the SMFA Function Index of about 7 points [23, 24]. The MID was derived using the distribution-based 0.5 SD method. Likewise, these MID values did not directly relate to what patients consider important. In addition, the difference between minimal important difference and minimal important change should be noted. De Vet et al. pointed out that a minimal important *change* regards to a longitudinal intra-individual process [20]. In contrast, a minimal important *difference* regards to a cross-sectional difference in scores between groups. Both values are not directly interchangeable, though there are methods to compare groups of patients using MIC values [20]. Currently, an anchor-based MID value is not known for the SMFA.

### Uncertainty around the MIC value

De Vet et al. pointed out that MIC values applied at the individual level, carry three types of uncertainty [20]. The first is the 95% confidence interval around the MIC. The MIC values in the present study had moderate to wide 95% confidence intervals, indicating that there is a moderate to large range in which the ‘true’ MIC value may fall. This may be caused by heterogeneity in injury type and severity in the study sample [3, 21].

A second form of uncertainty that should be evaluated is the proportion of patients for which application of the MIC would lead to an incorrect conclusion (e.g., misclassification) [20]. Some patients reported ‘no change,’ while their change in SMFA-NL score was actually *larger* than the MIC value. There are no clear guidelines what on what rate of misclassification and AUC are considered acceptable for MIC values; however, it is clear that low misclassification rates and high AUCs are preferred. We considered that especially the Upper Extremity Dysfunction and Mental and Emotional Problems subscales suffered from considerable misclassification (43% and 37% misclassification, respectively).

Within the research field of trauma surgery, high-quality studies that evaluated MIC values including a measure of accuracy are scarce. In a high-quality study of Pan et al., MIC values of the SF-36 and Lower Extremity Functional Scale (LEFS) were evaluated in patients with lower extremity injuries [25]. They reported AUC values ranging from 0.62 to 0.70. Mahabier et al. evaluated the Disabilities of the Arm shoulder and Hand (DASH) questionnaire in patients that sustained humeral shaft fractures and reported an AUC of 0.66 [26]. The MIC values that were reported in the present study showed a similar to slightly better accuracy (AUCs ranging from 0.63 to 0.78), compared to the MIC values of the SF-36, LEFS and DASH questionnaires.

Remarkably, MIC values that have been reported in trauma patients consistently showed a considerable inaccuracy, while the instruments have shown good clinimetric properties and employed study methods were adequate [25, 26]. This suggests that in trauma patients, there may be a relatively wide individual spread in what change in physical functioning is actually considered important by patients. This may possibly limit the ability of drawing a clear line that determines what change is important and what change is not. However, this hypothesis requires additional research to be confirmed.

A third form of uncertainty concerns the relation between MIC values and the measurement error of the instrument. Measurement error can be expressed as the smallest detectable change. When the SDC is smaller than the MIC value, importance of changes of individual patients can be distinguished from measurement error with at least 95% certainty [11, 20]. This was applicable to the *Lower Extremity Dysfunction* and *Problems with Daily Activities* subscales (Fig. 1). When the SDC is larger than the MIC, important changes may still be distinguished from measurement error, but the level of confidence is lower than 95%. The level of confidence can be obtained calculating the z value in the relation of the change in score to the standard error of measurement (SEM) using change in score = *z* value * √2 * SEM. SEM values of the SMFA-NL have been published in a similar population [15]. For example, on the *Mental and Emotional Problems* subscale the level of confidence at the MIC value is 59% (SEM = 5.95, *z* = 0.83). The MIC being smaller than the SDC limits the interpretability of individual changes on the *Upper Extremity Dysfunction* and *Mental and Emotional Problems* subscales at the individual level.

### Strengths and limitations

To the best of our knowledge, this is the first study that reported MIC values for the SMFA questionnaire, thereby increasing its interpretability. The MIC values were calculated using a logistic regression-based method that yields more reliable MIC values than the receiver operator characteristic-based method [18, 27]. However, there are several limitations that should be addressed. There was a substantial loss to follow-up. Patients that were not admitted to the hospital were less likely to respond to the follow-up questionnaire (data not shown). Patients with less severe injuries may already have recovered at the end of the interval. This may have led to follow-up bias, in which patients with less severe injuries were underrepresented. De Vet et al. showed that a more severe condition requires a greater improvement in score to be considered important to patients, yielding a larger MIC value [21]. Hence, the MIC may have been overestimated in the present study.

Few patients reported important deterioration, and therefore a deterioration-specific MIC value could not be calculated. Additionally, the Upper Extremity Dysfunction MIC value was calculated in a sample that showed a floor effect. Patients that caused a floor effect cannot improve in score, but may still have experienced important improvement. Therefore the MIC of the *Upper Extremity Dysfunction* subscale may be underestimated. This may also be an explanation of the profound misclassification of this subscale. Floor effects appear to be a specific limitation of the *Upper Extremity Dysfunction* subscale, since floor effects of this subscale have repeatedly been observed in the cross-cultural adaptation and validation studies of the SMFA-NL [13].

The GRE questions, which were used as an external criterion to determine the importance of the change that patients experienced, may also be considered a limitation. Although GRE questions are frequently used to calculate anchor-based MIC values, they have been criticized for its measurement performance and susceptibility to recall bias [28, 29]. There is increasing evidence that recall through a GRE question in musculoskeletal disorders is influenced by patients’ current functional status [28, 29]. This effect exaggerates when measurement intervals become longer [28]. Though not verifiable, such effects may have operated in the present study, considering the level of misclassification. An additional limitation of an anchor-based MIC is that it does not take measurement error into account. However, we have accounted for measurement error by comparing each MIC value to the SDC.

The novel MIC values of the SMFA-NL defined the smallest change in score on each subscale that can be considered important to patients. Whether occurred change is important to patients can be evaluated with acceptable levels of certainty (> 95%) on the *Lower Extremity Dysfunction* and the *Problems with Daily Activities* subscales. At the individual level, the *Upper Extremity Dysfunction* and the *Mental and Emotional Problems* subscale MIC values should be used with caution due to the level of misclassification and measurement error of the scales.

## Appendix 1

Global rating of effect (GRE) questions

- (1) How much problems do you currently have with using your (affected) arm(s), in comparison with 6 weeks after the injury?
- (2) How much problems do you currently have with walking, in comparison with 6 weeks after the injury?
- (3) How much problems do you currently have with performing your daily activities (such as self-care, doing groceries, labor, hobbies or household tasks), in comparison with 6 weeks after the injury?
- (4) How do you consider your current mental and emotional problems (such as anger, irritability, fatigue, concentration problems or sleeping problems), in comparison with 6 weeks after the injury?

## Appendix 2

Minimal important change scores for improvement: Slight improvement is important improvement

**Table:**

SMFA-NL subscales	*N*	Prop. Impr	Corr	MICpred	95% CI	MIC_adj_	Sens (%)	Spec (%)	%Mis	AUC
Upper extremity dysfunction	205	0.70	0.31	6	0.1–12.2	4	90	42	40	0.70
Lower extremity dysfunction	189	0.66	0.42	13	7.7–18.7	11	91	53	31	0.79
Problems with daily activities	182	0.85	0.26	25	12.7–41.0	20	94	27	34	0.72
Mental and emotional problems	188	0.59	0.25	4	−4.1–12.6	4	71	53	39	0.64

Adjusted minimal important change values of the SMFA-NL subscales were shown. All subscales use a 0–100 scale, in which 0 is best

*Prop. impr*. proportion of patients reporting improvement, *Corr* correlation of the anchor to the change-score, *MIC*_*pred*_ minimal important change for improvement, *MIC*_*adj*_ minimal important change for improvement adjusted for unequal proportions of improved/unchanged patients, *Sens* sensitivity, *spec* specificity, *%Mis* percentage misclassification, *AUC* area under the curve, *N.a*. not available, interval was too wide
